# Anthracycline-induced left atrioventricular dyssynchrony in children with leukemia: a cardiac magnetic resonance imaging study of functional and strain analysis

**DOI:** 10.3389/fcvm.2026.1874142

**Published:** 2026-07-29

**Authors:** Yi Liao, Yang Jia, Zhigang Yang, Linjun Xie, Shiyu Xiong, Yilei Wang, Xiangao Lei, Yingkun Guo, Rong Xu

**Affiliations:** 1 Department of Radiology, Key Laboratory of Birth Defects and Related Diseases of Women and Children of Ministry of Education, West China Second University Hospital, Sichuan University, Chengdu, Sichuan, China; 2 Department of Radiology, State Key Laboratory of Biotherapy, West China Hospital, Sichuan University, Chengdu, Sichuan, China

**Keywords:** anthracyclines, left atrial strain, left atrioventricular coupling index, left ventricular strain, magnetic resonance imaging

## Abstract

**Background:**

The 2022 European Society of Cardiology on cardio-oncology guidelines emphasized anthracycline chemotherapy's dose-dependent cardiotoxicity, identifying reduced left ventricular (LV) strain as a sensitive biomarker for early myocardial injury. However, left atrial (LA) structural/functional changes assessed via cardiac magnetic resonance (CMR) remain underexplored.

**Methods:**

This cross-sectional cohort study included 109 pediatric leukemia patients (2015–2021) and 40 age-matched controls. Leukemia patients were stratified into low-/high-dose anthracycline groups. CMR parameters (LA ejection fraction [LAEF], LV strain [LVS], LA strain [LAS], and left atrioventricular coupling index [LACI]) were analyzed. Linear regression evaluated strain-LACI relationships; Spearman's correlation assessed LAS-LVS associations.

**Results:**

Leukemia patients exhibited higher LA contraction strain (Ɛa: 24% vs. 21%, *p* = 0.025) but lower total/passive LAEF [73 ± 8 vs. 78 ± 7; 50 [40,56] vs. 59 [53,65]] and higher LV ejection fraction (64.5 ± 5.5 vs. 62.4 ± 5.7) than that in controls. LAS-LVS correlations were stronger in controls (r = 0.32–0.52) than leukemia (r = 0–0.27). Among leukemia patients, low-dose patients had higher active LAEF (48 ± 14 vs. 39 ± 16) and weaker LAS-LVS correlations than high-dose group(r = 0–0.28 vs. r = 0–0.42), while high-dose group showed elevated LACI (*β*=9.68, *p* = 0.018) correlating with the cumulative anthracycline dose.

**Conclusions:**

Anthracyclines disrupt LA-LV coordination in pediatric leukemia, with higher doses exacerbating atrial strain impairment. These findings highlight CMR-derived strain parameters as critical for monitoring anthracycline-induced cardiac impairment.

## GRAPHICAL ABSTRACT

**Figure:**
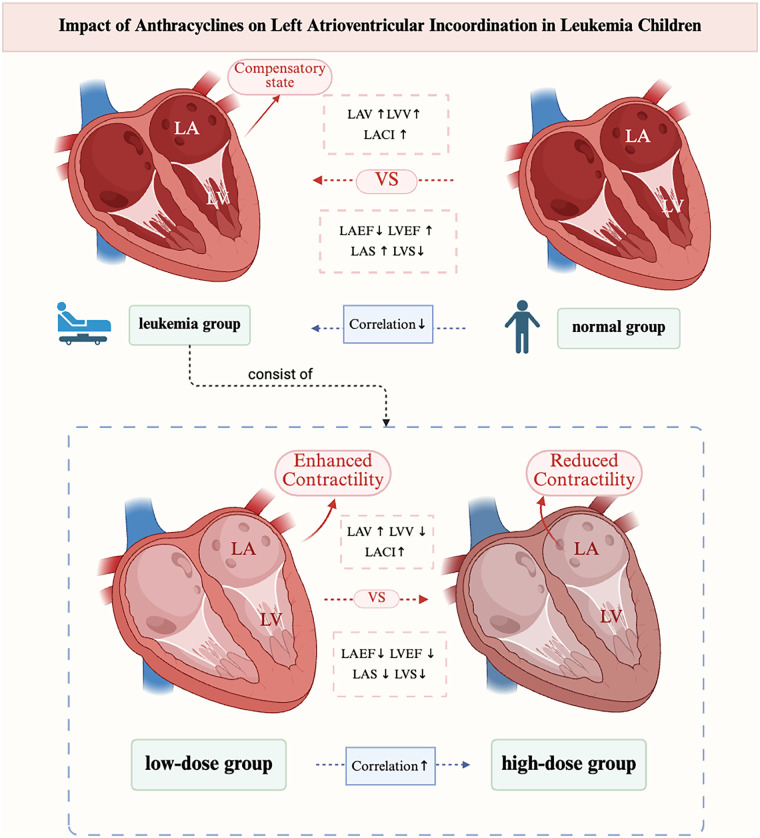
LAV: left atrial volume; LVV: left ventricular volume; LACI: Left atrioventricular coupling index; LAS: left atrial strain; LVS: left ventricular strain; Correlation: correlation between LAS and LVS.

## Introduction

Anthracyclines are the most widely chemotherapeutic drugs used in the frontline of cancer treatment, which may cause cardiovascular toxicity and lead to long-term adverse outcomes (1, 2). According to the 2022 ESC (European Society of Cardiology) Guidelines on cardio-oncology, abnormalities in left ventricular (LV) function and structure-particularly reduced global longitudinal strain (GLS)-are recognized as early indicators of cancer therapeutics-related cardiac dysfunction (CTRCD) and are closely linked to subsequent heart failure (3). Leukemia is the most common type of disease among children and anthracyclines constitute a key component in leukemia therapy (4). But are associated with dose-dependent late-onset cardiac toxicity, leading to morbidity and mortality among leukemia survivors in childhood (5, 6).

Anthracyclines may induce myocardial injury through multiple direct and indirect pathways, potentially affecting global cardiac function (7), including left atrium (LA) and LV. The cardiotoxic effects of anthracyclines exhibit a dose-dependent relationship, with higher doses significantly elevating the likelihood of myocardial damage than lower doses, potentially leading to decreased left ventricular ejection fraction (LVEF) and heart failure (3, 8). Our previous research showed that the GLS of the LV in children with leukemia decreased, suggesting that anthracycline drugs have an impact on the LV during childhood (9). While limited studies suggest possible left atrial (LA) abnormalities following anthracycline exposure (10–12), pediatric-specific data remain scarce. Elucidating LA remodeling could enhance understanding of the cardiotoxic profile of anthracyclines.

The left atrium and ventricle function as an integrated unit, and pathophysiological adaptations in one chamber may induce compensatory changes in the other—a concept termed atrioventricular interaction reported by other cardiac diseases (13–15). However, the role of atrioventricular interactions in anthracycline-induced cardiotoxicity remains poorly defined, particularly in pediatric populations. Prior studies in adult ALL survivors have identified weak correlations between left atrial strain (LAS) and GLS (16), but the influence of anthracycline dosage on these interactions was not explored. Moreover, the dynamics of atrioventricular coupling during active leukemia treatment in children are unclear. Feature-tracking cardiac magnetic resonance imaging (CMR) has emerged as a superior non-invasive modality for assessing LAS compared to echocardiography (17), offering enhanced precision in strain analysis. This study aims to investigate the impact of cumulative anthracycline exposure on left heart injury in pediatric acute leukemia patients during treatment using CMR feature-tracking. We hypothesize that the myocardial toxicity caused by anthracycline drugs has varying degrees of impact on the left atrium and left ventricle and will interfere with the coordination of atrioventricular interaction.

## Materials and methods

### Study population

This single-center cross-sectional study, conducted at our hospital, was part of a prospectively included research cohort. The study prospectively collected pediatric patients with leukemia who had a history of anthracycline drug treatment and had stopped the medication for no more than one-year, enrolling pediatrics patients who were diagnosed with leukemia from May 2015 to December 2021. The reasons for patients to undergo cardiac MRI included chest tightness, tachycardia, or other chest discomfort experienced during leukemia treatment. The study excluded patients who (1) had CMR images that were not of sufficient quality for analysis; (2) had any congenital heart disease, such as atrial septal defect, ventricular septal defect, hypertrophic cardiomyopathy, etc.; (3) had abnormal cardiac structure and function, such as cardiac enlargement and decreased cardiac function, reported in any ultrasound before the diagnosis of leukemia; and (4) were older than 18 years old (Supplementary Figure S1).

Healthy controls were prospectively enrolled through systematic recruitment (October 2019-March 2023) using public advertisements (18). Prior to CMR enrollment, parents completed screening questionnaires documenting clinical/family history to exclude children with systemic diseases or genetic disorders. Age-matched controls (normal group) were selected from this cohort based on age, gender, height, and weight parameters matching leukemia patients undergoing CMR **(**Supplementary Table S1**).** Exclusion criteria remained identical between groups, ensuring demographic and clinical comparability for comparative analysis.

This study was approved by the Biomedical Research Ethics Committee of our hospital. Written informed consent was obtained from all participants. Registration number: ChiCTR1800017054 http://www.chictr.org.cn

### Clinical data

All clinical data for the participants were collected from the medical records of our hospital or hand-written questionnaires. For all pediatric leukemia patients, the following clinical information was gathered: gender, leukemia type (ALL or AML), chemotherapy regimen (CCCG-ALL-2015–Chinese Children's Cancer Group 2015 protocol or other protocols), clinical risk grouping, FAB classification (French-American-British classification), immunology classification, fusion gene, and patient age, height, and weight at the time of CMR examination. Additionally, information on the treatment phase at examination, cumulative anthracycline dosage up to the time of examination, and the presence of sinus tachycardia or sinus arrhythmia during the period from initial diagnosis to examination was collected. Cumulative anthracycline doses were converted to doxorubicin equivalents using conversion factors: 0.6 for daunorubicin, 0.8 for epirubicin, and 10.5 for mitoxantrone (5). According to the European guidelines on cardio-oncology, patients exposed to a cumulative doxorubicin dose < 100 mg/m^2^ were classified as low risk. The remaining patients were classified as – moderate risk (3). Participants were grouped based on anthracycline dosage: low-dose group (<100 mg/m^2^), high-dose group (≥100 mg/m^2^), and a control group of normal children. The criteria for classifying patients into clinical risk groups, FAB classification, immunology classification, as well as the cumulative anthracycline dosage, followed the protocols for the treatment of these pediatric patients (19, 20). For healthy volunteers, gender, age, height, and weight were collected during the CMR examination via a questionnaire. Body mass index (BMI) was calculated by dividing weight by the square of height. Body surface area (BSA) was computed using the Xu Wensheng formula: BSA (m^2^) = 0.0061 × height (cm) + 0.0128 × weight (kg) −0.1529.

### MR protocol

Medical imaging technologists with 5 years of experience (YW and XL) conducted scans on these participants. All the population underwent CMR with the use of a supine, head-advanced mode with the use of a 1.5-Tesla magnetic resonance scanner (Philips Achieva, Philips, the Netherlands) with the use of a 16-channel body-coil. The acquisition signal was triggered by respiratory ECG gating in all children. For children who could cooperate with the examination, breath-hold mode was used for image acquisition. For children who could not cooperate with the examination, the examination was performed under a steady breathing state with sedatives or early sleep deprivation. The scanning sequence included cardiac cine imaging. The specific scanning conditions and parameters are as follows: Cardiac cine was performed with True FISP sequence, and the scanning protocol was according to the standard scanning protocol recommended by the International Society of Cardiac Magnetic Resonance. The scanning range covered the whole left ventricle (from the level above the mitral valve to the apical level of the heart), and 6–10 consecutive short-axis cine images completely covering the ventricle were obtained. The images of two-chamber, four-chamber and three-chamber heart were collected successively. The specific scanning parameters were as follows: repeat time TR: 43.94 ms, echo time TE: 1.48 ms, flip Angle: 50°, slice thickness: 6–8 mm; FOV: 213× 213–400 × 400, matrix: 133× 133–250 × 250. Twenty-five frames of images were acquired for each cardiac cycle. The sequence was scanned for approximately 3–5 min.

### Imaging analysis

Imaging diagnostic physicians LX and YJ, with 4 and 3 years of experience in CMR post-processing, respectively, performed LA myocardial feature tracking (FT) analysis using Cvi42 (cmr42, version 5.9.3; Circle Cardiovascular Imaging Inc., Canada). For quality control, all imaging analysis outcomes were reviewed by an experienced CMR radiologist with 8 years of experience ZY. These three physicians were completely unaware whether the traced images were from leukemia patients or normal controls, and they were also unaware of the corresponding clinical information.

### LA feature-tracking analysis

**Figure 1:**
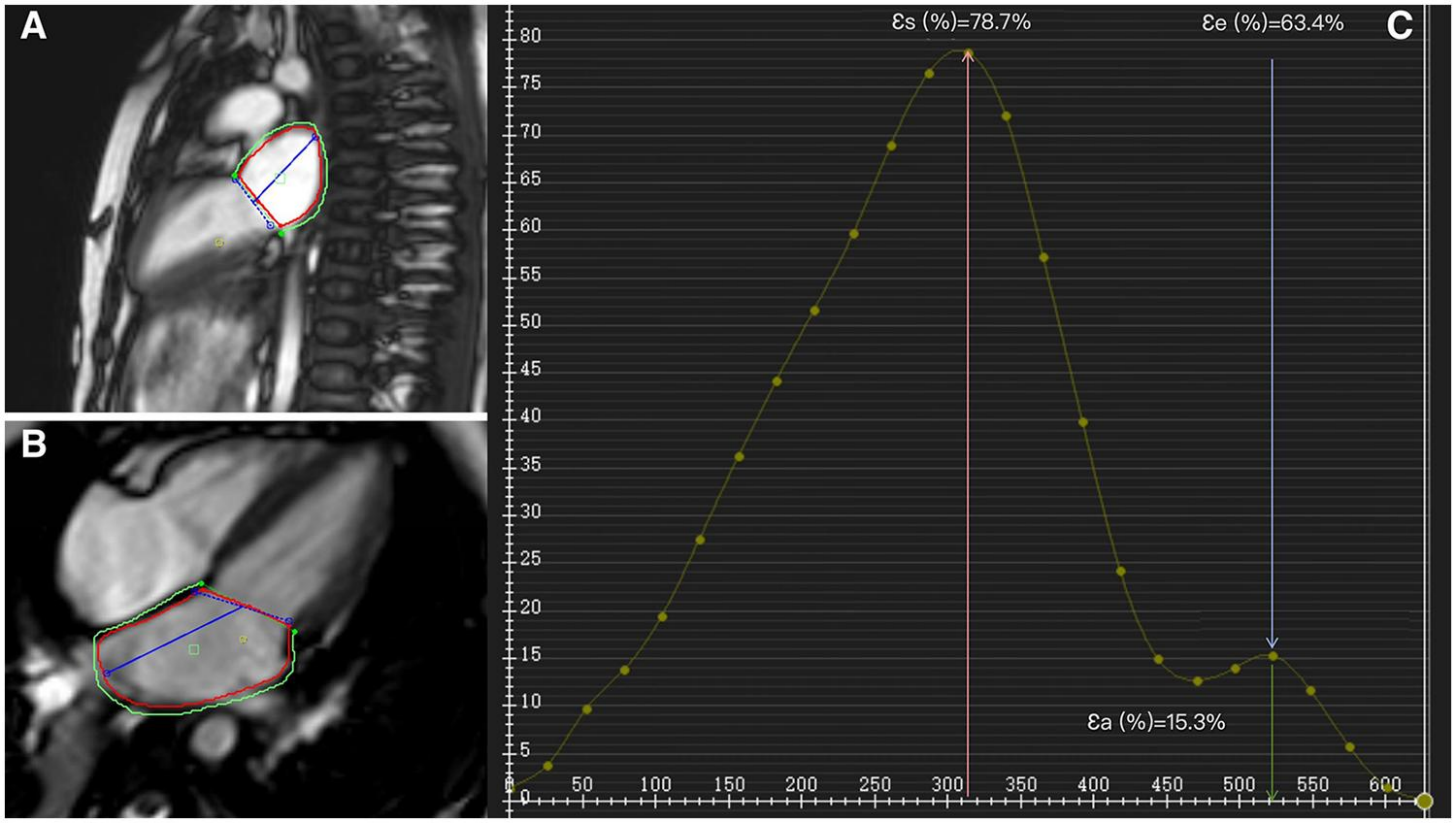
LA measurements by CMR feature tracking. **(A** and **B)** LA longitudinal strain in the two- and four-chamber views at end-diastole. **(C)** The LA strain and strain rate curve. The red line is endocardial curve, and the green line is epicardial curve. Global endocardial LA strain values were recorded. The red arrow points to Ɛs. The blue arrow points to Ɛe. The green arrow points to Ɛa. Ɛs, reservoir strain; Ɛe (Ɛs- Ɛa), conduit strain; Ɛa, booster pump strain.

The LA endocardial and epicardial boundaries were manually traced in the 2- and 4-chamber views using the point-click method with end-diastole as the reference period. The software then applies an automatic tracking algorithm to delineate the atrial boundary in subsequent frames. Three phases of LA overall longitudinal strain were analyzed, including LA reservoir strain (*ε*s), conduit strain (*ε*e) and booster pump strain (*ε*a), which was shown in Figure 1.The symbol “*ε*” indicates that the data should be taken as the absolute value.

### LA volumetric and emptying fraction analysis

Manual tracings of the LA length and area were performed in the 2-chamber and 4-chamber long-axis views using CVI software. LA volume parameters were evaluated using the validated biplane area length method and indexed to BSA according to a previously described formula (20). LA appendage and pulmonary veins were excluded from the LA volume. The volume indices of the LA during each phase were defined as the maximum LA volume (LAVmax) at LV end-systole (just before mitral valve opening), minimum LA volume (LAVmin) at LV end-diastole (just after mitral value closing), and pre-atrial contraction LA volume (LAVpreA) before the initiation of atrial contraction (or the last frame before mitral valve reopening) (21).

The total LA emptying fraction (LA total, corresponding to atrial reservoir function) was calculated as 100%*(LAV-max ₋ LAV-min)/LAV-max. The passive LA emptying fraction (LA passive, corresponding to atrial conduit function) was calculated as 100%*(LAV-max ₋ LAV-preA)/LAV-max. The active LA emptying fraction (LA active, corresponding to atrial contractile pump function) was calculated as 100%*(LAV-preA ₋ LAV-min)/LAV-preA.

### LV function and feature-tracking analysis

To estimate ventricular volumes and functions, we determined the left ventricular end-diastolic volume (LVEDV) and end-systolic volume (LVESV) by tracing the endocardial borders at the respective phases. The left ventricular stroke volume (LVSV) was calculated as the difference between LVEDV and LVESV. Left ventricular cardiac output (LVCO) was then obtained by multiplying LVSV with the heart rate. The left ventricular ejection fraction (LVEF) was computed as the ratio of LVSV to LVEDV. Additionally, myocardial strain analysis was performed using feature tracking (FT) technology with cine imaging. At the end of diastole, the endocardial and epicardial borders were automatically traced and manually corrected. LV two-dimensional (2D) global radial, circumferential, and longitudinal strains were automatically derived. Short-axis images were analyzed to obtain global circumferential strain (GCS) and global radial strain (GRS). Global longitudinal strain (GLS) was measured using long-axis images.

### Left atrioventricular coupling Index

Left atrioventricular coupling index (LACI) was defined as the ratio of the left atrial minimum volume at the end of diastole (LAVmin) to the left ventricular end-diastolic volume (LVEDV), expressed as a percentage. Higher values indicated a relatively larger atrial volume compared to ventricular volume, representing a more “abnormal” state (22).

### Reproducibility

A total of 30 subjects were randomly selected, including 15 leukemia children and 15 normal children. The intra-observer and inter-observer reproducibility of LA and LV parameters were analyzed. To evaluate the inter-observer agreement of measurements, two investigators independently performed measurements on the above subjects without knowledge of the clinical data of group. To evaluate intra-observer agreement, one of the investigators performed two measurements on the same subjects in two different months.

### Statistical analysis

Categorical variables are expressed as counts (percentages), while continuous variables are reported as mean ± standard deviation or median [interquartile range], depending on their distribution, which was determined using the Shapiro–Wilk test and QQ plots. Groups of participants were compared using the chi-square test or Fisher's exact test for categorical data and the Student's t-test or Mann–Whitney U test for continuous data, depending on the nature of the data. Analysis of Kruskal–Wallis rank sum test were used to compare the differences in left atrial and left ventricular parameters between groups. Given the inherent age differences between the low-dose and high-dose groups, we employed multivariable linear regression models adjusting for age to evaluate the independent effects of dosage groups on LAS, LVS, and LAEF. We analyzed the correlation between LAS and LVS using a Spearman heatmap, where red signifies positive correlation (intensity reflected strength) and blue-to-red gradient indicated negative to positive shift. Univariate linear regression analyses were performed to investigate the relationship between clinical variables and LACI. Simultaneously, both univariate linear regression analyses were conducted to examine the factors (LAS and LVS) influencing LACI.

Intraobserver and interobserver agreement were assessed by Bland-Altman analysis and intraclass correlation coefficients (ICC). ICC estimates and their 95% confidence interval were based on a single-measure, two-way mixed (consistency) model (< 0.5: poor, 0.5–0.75: moderate; 0.75–0.9: good, > 0.9: excellent).

In our study, all statistical analyses were performed using R software (version 4.2.2, R Foundation for Statistical Computing, Vienna, Austria), and Prism GraphPad Version 10.3.0 (461) for graphical representation. P values were two-sided, and a *P* < 0.05 was considered statistically significant.

## Results

### Participant characteristics

In this study, a total of 109 pediatric leukemia patients and 40 normal children were included, as shown in Supplementary Figure S1. The CMR examinations of these children with leukemia were conducted from October 2017 to October 2022. No statistically significant differences were observed between the normal group and the leukemia group regarding age (*p* = 0.489), BMI (*p* = 0.219), or sex distribution (*p* = 0.403), as presented in Supplementary Table S1.

Leukemia patients were categorized into two groups based on the cumulative dose of anthracycline drugs: the low-dose group (<100 mg/m^2^; *n* = 83, 76.1%) and the high-dose group (≥100 mg/m^2^; *n* = 26, 23.9%). Statistically significant differences (*p* < 0.05) were observed between the groups for several key variables. Participants in the high-dose group were significantly older at examination (median 12.7 years, IQR: 10.0, 14.2) compared to those in the low-dose group (median 6.6 years, IQR: 4.4, 8.5; *p* < 0.001) and had a larger body surface area (BSA) (median 1.22 m^2^, IQR: 1.12, 1.38 vs. 0.83 m^2^, IQR: 0.66, 0.96; *p* < 0.001). The distribution of clinical risk groups differed markedly, with a significantly greater proportion of patients classified as intermediate/high risk in the high-dose group (96.2%) compared to the low-dose group (51.8%; *p* < 0.001). Furthermore, a positive fusion gene status was more prevalent in the high-dose group (46.2%) than in the low-dose group (21.7%; *p* = 0.015). No statistically significant differences were found between the groups for BMI at examination (*p* = 0.834), interval time in weeks (*p* = 0.949), sex distribution (*p* = 0.126), treatment phase (*p* = 0.809), disease classification (*p* > 0.999), FAB classification (*p* = 0.774), immunophenotype (*p* = 0.062), chemotherapy regimen (*p* = 0.677), or the presence of an abnormal ECG (*p* = 0.831), as further detailed in Table 1.

**Table 1:** Basic clinical characteristics of the low-dose group and the high-dose group.

Characteristic	group		p-value
	low-dose group *N* = 83^a^	high-dose group *N* = 26^a^	
**Age at examination**	6.6 (4.4, 8.5)s	12.7 (10.0, 14.2)	<0.001^b^
**BMI at examination**	17.1 (15.7, 19.2)	17.4 (15.2, 21.4)	0.834^b^
**BSA at examination**	0.83 (0.66, 0.96)	1.22 (1.12, 1.38)	<0.001^b^
**Interval time (weeks)**	62 (35, 94)	49 (37, 98)	0.949^b^
**Sex**			0.126^c^
Male	40 (48.2%)	17 (65.4%)	
Female	43 (51.8%)	9 (34.6%)	
**Clinical risk groups**			<0.001^c^
Low risk	40 (48.2%)	1 (3.8%)	
Intermediate/high risk	43 (51.8%)	25 (96.2%)	
**Treatment phase examination**			0.809^c^
Treatment phase ˂54weeks	35 (42.2%)	12 (46.2%)	
54 weeks ≤ treatment phase ≤125weeks	28 (33.7%)	7 (26.9%)	
Treatment phase ˃ 125weeks	20 (24.1%)	7 (26.9%)	
**Disease classification**			>0.999^d^
ALL	78 (94.0%)	25 (96.2%)	
AML	5 (6.0%)	1 (3.8%)	
**FAB classification**			0.774^d^
L1	21 (25.3%)	5 (19.2%)	
L2	55 (66.3%)	18 (69.2%)	
Other	7 (8.4%)	3 (11.5%)	
**Immunophenotyping**			0.062^d^
early pre B	41 (49.4%)	10 (38.5%)	
pre B	25 (30.1%)	4 (15.4%)	
B	7 (8.4%)	3 (11.5%)	
T	2 (2.4%)	3 (11.5%)	
Other	8 (9.6%)	6 (23.1%)	
**Fusion gene**			0.015^c^
negative	65 (78.3%)	14 (53.8%)	
positive	18 (21.7%)	12 (46.2%)	
**Chemotherapy regimen**			0.677^d^
CCCG-ALL-2015	76 (91.6%)	25 (96.2%)	
Other	7 (8.4%)	1 (3.8%)	
**Abnormal ECG**			0.831^c^
no	30 (36.1%)	10 (38.5%)	
yes	53 (63.9%)	16 (61.5%)	

**low-dose group:** dosage of anthracycline drugs ˂100 mg/m^2^; **high-dose group:** dosage of anthracycline drug ≥100 mg/m^2^.

**Interval time (weeks): t**he interval between the diagnosis of leukemia and the cardiac MRI examination in pediatric patients with leukemia.

**Abnormal ECG:** sinus tachycardia or sinus arrhythmia during the period from initial diagnosis to examination.

BMI, body mass index; BSA, body surface area; ALL, acute lymphocyte leukemia; AML, acute myelocytic leukemia; FAB classification, French-American-British classification; CCCG-ALL-2015, Chinese Children's Cancer Group 2015 protocol; ECG, electrocardiographic.

a Median (Q1, Q3); n (%).

b Wilcoxon rank sum test.

c Pearson's Chi-squared test.

d Fisher's exact test.

### CMR volume and strain parameters difference between normal and leukemia groups

Regarding LA parameters, participants in the leukemia group exhibited significantly higher values for LA Ɛa (median 24%, IQR: 19, 30 vs. median 21%, IQR: 16, 26; *p* = 0.025), while the leukemia group demonstrated significantly lower values for left atrial volume index - LAVmax I (median 23, IQR:18, 30 vs. median 29,IQR:25, 32; *p* = 0.013), LA total (73 ± 8% vs. 78 ± 7%; *p* < 0.001), and LA passive (median 50%, IQR: 40, 56 vs. median 59%, IQR: 53, 65; *p* < 0.001).

For LV parameters, participants in the leukemia group showed significantly greater values for LVEF (64.5 ± 5.5% vs. 62.4 ± 5.7%; *p* = 0.049). HR in the leukemia group was expressively lower than that in the normal group (85 ± 16 vs. 92 ± 15; *p* = 0.024).

LACI was of no statistical significance between the two groups though, there was a upward trend in the leukemia group median 8.9, IQR: 7.1, 11.2 vs. median 8.4, IQR: 6.3, 11.0, as shown in Table 2.

**Table 2:** Comparison of LAS, LVS and conventional magnetic resonance parameters and strain in the normal and leukemia groups.

Characteristic	Group		p-value
	Leukemia *N* = 109^a^	Normal *N* = 40^a^	
**HR**	85 ± 16	92 ± 15	**0**.**024**^c^
**LACI (%)**	8.9 (7.1, 11.2)	8.4 (6.3, 11.0)	0.492^b^
**LA function and strain parameters**			
**LA Ɛs (%)**	73 (59, 82)	71 (63, 78)	0.367^b^
LA Ɛe* (%)	47 (39, 56)	51 (44, 55)	0.293^b^
LA Ɛa* (%)	24 (19, 30)	21 (16, 26)	**0**.**025**^b^
**LAVmax I**	23 (18, 30)	29 (25, 32)	**0**.**013**^b^
**LAVmin I**	6.35 (4.73, 8.00)	5.59 (4.12, 6.99)	0.128^b^
**LAVpre-A I**	12.4 (9.6, 15.2)	10.3 (8.6, 13.4)	0.126^b^
**LA total (%)**	73 ± 8	78 ± 7	**<0**.**001**^c^
**LA passive (%)**	50 (40, 56)	59 (53, 65)	**<0**.**001**^b^
**LA active (%)**	46 ± 15	48 ± 13	0.341^c^
**LV function and strain parameters**			
**LVEDVI**	70 (61, 78)	67 (59, 76)	0.156^b^
**LVESVI**	25.1 (21.2, 29.1)	24.6 (20.8, 28.6)	0.701^b^
**LVSVI**	45 (38, 51)	42 (35, 48)	0.054^b^
**LVCOI**	3.73 (3.26, 4.27)	3.76 (3.24, 4.27)	0.968^b^
**LVEF(%)**	64.5 ± 5.5	62.4 ± 5.7	**0**.**049**^c^
LVGLS* (%)	19.44 (17.50, 21.08)	20.50 (18.06, 21.12)	0.242^b^
**LVGRS (%)**	36 (32, 42)	38 (30, 41)	0.961^b^
LVGCS* (%)	19.90 ± 1.86	20.08 ± 1.57	0.559^c^

LACI, Left atrioventricular coupling index; LA Ɛs, reservoir strain; LA Ɛe, conduit strain; LA Ɛa, contraction strain; LAVmax I, the maximum left atrial volume index; LAVmin I, minimum left atrial volume index; LAVpre-A I, pre-atrial contraction left atrial volume index; LA total, total left atrial emptying fraction; LA passive, passive left atrial emptying fraction; LA active, active left atrial emptying fraction; LVEDVI, left ventricular end-diastolic volume index; LVESVI, left ventricular end- systolic volume index; LVSVI, left ventricular stroke volume index; LVCO, left ventricular cardiac output; LVEF, left ventricular ejection fraction; LVGLS, global longitudinal strain; LVGRS, global radial strain; LVGCS, global circumferential strain.

a Median (Q1, Q3); Mean ± SD

b Wilcoxon rank sum test

c Welch Two Sample t-test

d For the values of LA Ɛe, LA Ɛa, LVGLS and LVGCS, take their absolute values.

The bolded values indicate that the *p*-values are less than 0.05, which means they are statistically significant.

When the leukemia group was stratified into low-dose and high-dose subgroups (presented in Supplementary Table S2), we observed that the low-dose group exhibited significantly higher LA Ɛa compared to the normal control group (median 25%, IQR: 20, 30 vs. median 21%, IQR: 16, 26; *p* = 0.010). No statistically significant difference in LA Ɛa was detected between the high-dose group and normal controls. LA active was significantly reduced in the high-dose group vs. normal controls (39 ± 16% vs. 48 ± 13%; *p* = 0.020), whereas no such difference was observed between the low-dose group and normal controls. Notably, a trend toward statistical significance was noted for LVGLS when comparing the high-dose group to normal controls (median 17.94%, IQR:15.94, 21.14 vs. median 20.50%, IQR:18.06, 21.12; *p* = 0.055). This trend was not present when comparing the low-dose group to normal controls.

### CMR volume and strain parameters difference between low-dose and high-dose groups

Statistically significant differences were noted for lower LA active (39 ± 16% vs. 48 ± 14%; *p* = 0.018) and lower LVCOI(median 3.33, IQR: 2.94, 4.13 vs. median 3.81, IQR 3.37, 4.35; *p* = 0.017) in the high-dose group. After adjusting for age, the association between dose groups and LA active was not statistically significant; however, the low-dose group showed a trend toward higher LA active values compared to the high-dose group (*β* = 7.10; 95% CI, −0.35 to 14.56; *P* = 0.065) **(**Supplementary Table S3**)**. LACI was of no statistical significance between the two groups though, there was a upward trend in the high-dose group, median 10.0%, IQR: 6.4, 12.3 vs. median 8.9%, IQR: 7.1, 10.9, as shown in Table 3.

**Table 3:** Comparison of LAS, LVS and conventional magnetic resonance parameters and strain in the low-dose and high-dose groups.

Characteristic	Group		p-value
	High-dose group *N* = 26^a^	Low-dose group *N* = 83^a^	
**HR**	81 ± 19	87 ± 15	0.160^c^
**LACI (%)**	10.0 (6.4, 12.3)	8.9 (7.1, 10.9)	0.511^b^
LA function and strain parameters			
**LA Ɛs (%)**	65 (57, 78)	75 (60, 83)	0.107^b^
**LA Ɛe**^d^ **(%)**	44 (39, 49)	49 (37, 57)	0.155^b^
**LA Ɛa**^d^ **(%)**	22 (16, 27)	25 (20, 30)	0.114^b^
**LAVmax I**	20 (17, 27)	24 (20, 31)	0.110^b^
**LAVmin I**	6.64 (4.50, 9.81)	6.33 (4.87, 7.78)	0.940^b^
**LAVpre-A I**	9.8 (7.7, 15.5)	12.6 (10.1, 15.2)	0.120^b^
**LA total (%)**	72 (64, 75)	74 (69, 77)	0.086^b^
**LA passive (%)**	52 (39, 61)	49 (40, 56)	0.420^b^
**LA active (%)**	39 ± 16	48 ± 14	**0**.**018**^c^
LV function and strain parameters			
**LVEDVI**	68 (62, 75)	72 (60, 79)	0.323^b^
**LVESVI**	24.4 (22.2, 28.8)	25.1 (20.5, 29.3)	>0.999^b^
**LVSVI**	44 (37, 48)	46 (40, 51)	0.196^b^
**LVCOI**	3.33 (2.94, 4.13)	3.81 (3.37, 4.35)	**0**.**017**^b^
**LVEF(%)**	63.7 ± 6.5	64.7 ± 5.1	0.480^c^
**LVGLS**^d^ **(%)**	17.94 (15.94, 21.14)	19.55 (18.07, 21.08)	0.071^b^
**LVGRS (%)**	34 (26, 42)	37 (34, 41)	0.107^b^
**LVGCS**^d^ **(%)**	19.85 ± 2.02	19.91 ± 1.82	0.888^c^

Abbreviations as in Table 2.

a Median (Q1, Q3); Mean ± SD.

b Wilcoxon rank sum test.

c Welch Two Sample t-test.

d For the values of LA Ɛe, LA Ɛa, LVGLS and LVGCS, take their absolute values.

The bolded values indicate that the *p*-values are less than 0.05, which means they are statistically significant.

In the subgroup analysis of the high-dose group, the LACI was higher in the subgroup under medication than in the subgroup with drug discontinuance, median 10.7%, IQR: 8.1, 14.2 vs. median 6.1%, IQR: 4.0, 9.3, *p* = 0.013 (Supplementary Table S4).

### LACI and the cumulative dose of anthracyclines

**Figure 2:**
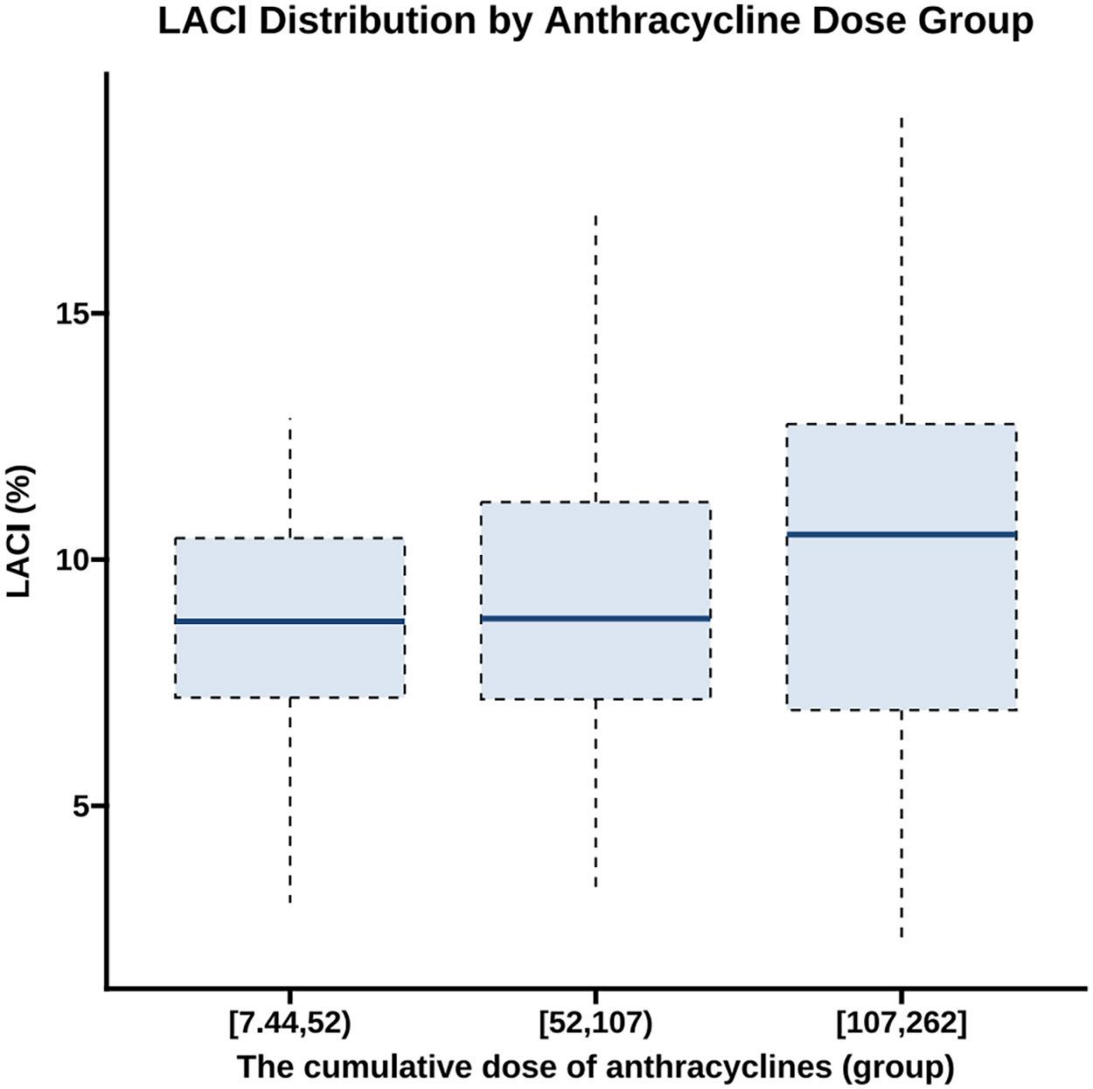
Box plot illustrating the distribution of LACI across three anthracycline dose groups, as determined by K-means clustering. Leukemia patients stratified by anthracycline cumulative dose (mg/m^2^): [7.44,52), [52,107), [107,262].

Based on the cumulative dose of anthracyclines, patients with leukemia were stratified into three groups using K-means clustering: [7.44, 52), [52, 107), and [107, 262] mg/m^2^, as illustrated in the box plot (Figure 2). The group with the highest anthracycline cumulative dose [(107, 262) mg/m^2^] exhibited elevated LACI values, although this difference did not reach statistical significance. Additionally, Table 4 demonstrates that there was no statistically significant linear correlation between the cumulative dose of anthracyclines and LACI values in low-dose group. However, LACI was correlated to the increasing cumulative dose of anthracyclines in high-dose group (*β* = 9.68; 95% CI, 2.22 to 17.14; *p* = 0.018).

**Table 4:** Univariate linear regression analysis of influencing factors for LACI in low-dose and high-dose group patients respectively.

Characteristic	Low-dose group (*N* = 83)			High-dose group (*N* = 26)		
	Beta	95% CI	p-value	Beta	95% CI	p-value
**Age at examination**	0.71	−1.04, 2.46	0.428	6.93	−2.69, 16.55	0.171
**BMI at examination**	−0.78	−3.94, 2.39	0.632	2.82	−5.58, 11.22	0.517
**BSA at examination**	0.45	−2.38, 3.28	0.757	17.04	3.57, 30.50	**0**.**021**
**The cumulative dose of anthracyclines (mg/㎡)**	0.30	−0.95, 1.55	0.639	9.68	2.22, 17.14	**0**.**018**

CI, confidence interval; BMI, body mass index; BSA, body surface area.

All continuous independent variables were subjected to logarithmic transformation.

The bolded values indicate that the *p*-values are less than 0.05, which means they are statistically significant.

### Association between LAS and LVS

**Figure 3:**
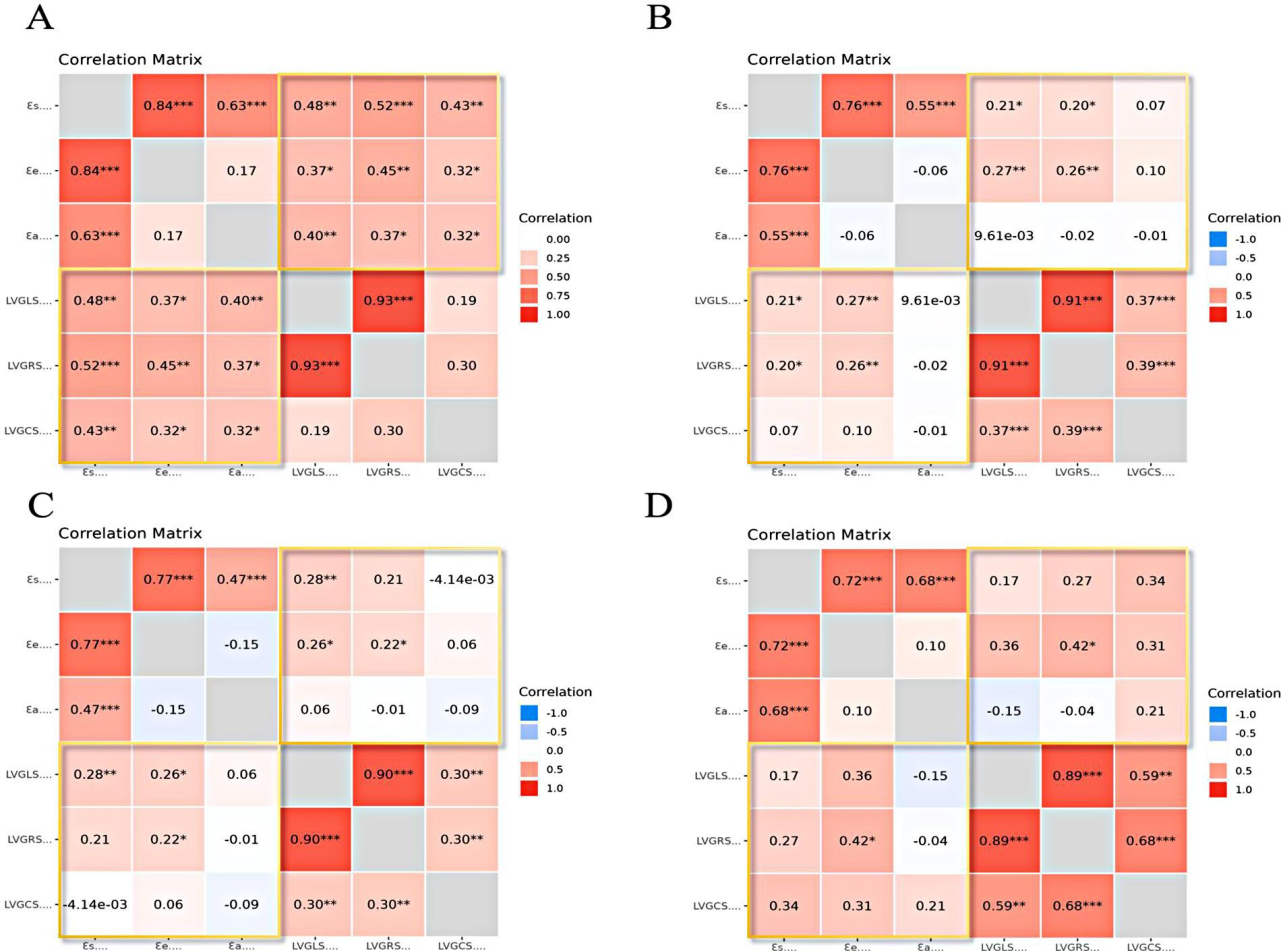
Spearman correlation of LAS and LVS in leukemia and normal children. The regions marked by orange borders highlight the correlation results among the variable groups. **(A)** Spearman correlation of LAS and LVS in normal children. **(B)** Spearman correlation of LAS and LVS in leukemia children. **(C)** Spearman correlation of LAS and LVS in Low-dose group. **(D)** Spearman correlation of LAS and LVS in high-dose group. Red areas denote significant positive correlations, with color intensity reflecting correlation strength; blue-to-red gradient indicates transition from negative to positive correlation. Abbreviations as in Table 4. *p < 0.05, **p < 0.01, ***p < 0.001.

In the normal control group, a strong positive correlation was observed between left atrial strain (LAS) and left ventricular strain (LVS), with correlation coefficients ranging from 0.32 to 0.52. All correlations were statistically significant, as illustrated in Figure 3A. Conversely, in the leukemia group, a weak correlation was found between left ventricular global longitudinal strain (LVGLS) and LA reservoir strain (LA *ε*s), with a Spearman correlation coefficient of only 0.21 (*p* < 0.05). This might suggest that under specific physiological or pathological conditions, the contribution or influence of LA *ε*s on overall LVGLS is relatively limited, as depicted in Figure 3B. In the low-dose group, LVGLS and LA *ε*s exhibited a weak correlation with a coefficient of 0.28 (*p* < 0.01), as shown in Figure 3C. In the high-dose group, the correlation between LAS and LVS was not statistically significant. However, a weak negative trend (−0.15) was noted between LVGLS and LA pump strain (LA *ε*a) within this group, as visualized in Figure 3D.

In the high-dose group, there was no statistically significant correlation between LAS and LVS in either the group currently undergoing treatment or the group that had discontinued treatment; however, a trend in the correlation between ɛs and LVGLS was observed to be reversed depending on the treatment phase (Supplementary Figure S2).

### Reproducibility

The intra-observer and inter-observer reproducibility of LAS and LVS were considered excellent (all ICCs >0.75 respectively). The intra-observer and inter-observer correlation coefficients are shown in Supplementary Table S4. Bland-Altman analyses revealed minimal systematic biases in most measurement comparisons which were presented in Supplementary Figures S3, S4.

## Discussion

In this cross-sectional study, we observed inconsistent changes in left atrial and ventricular volume and strains in childhood leukemia patients treated with anthracycline. Key findings include higher left atrial booster pump strain(LA Ɛa) in leukemia patients but relatively lower left ventricular strain than the normal control group. LAEF(total, passive, active) was lower in leukemia patients but LVEF was higher. However, LA strains(LAS) and LV strains(LVS) were all at lower trend in the high-dose group than in the low-dose when comparing among leukemia patients. So did LAEF and LVEF trend among leukemia patients. In normal children, LAS and LVS were highly correlated, but this correlation was low in leukemia patients. High-dose groups showed a stronger LAS-LVS correlation than low-dose groups. We hypothesize that anthracycline treatment initially enhances left atrial contractility, leading to an increase in LAS as a compensatory mechanism for the mild decline in left ventricular diastolic function. However, with the accumulation of anthracycline dosage, this compensatory effect gradually diminishes, manifested by decreases in both LAS and LVS.

### Changes in left atrial strain in pediatric leukemia patients with different cumulative dosage groups

Previous studies have suggested that LA Ɛs, LA Ɛe, and LA Ɛa reflect the deformation capabilities of the left atrium during ventricular systole (reservoir function), early ventricular diastole (conduit function), and late ventricular diastole (booster pump function), respectively (23). Fernández-Avilés, Consuelo et al. found that survivors of childhood acute lymphoblastic leukemia (ALL) exhibited a decrease in both LA Ɛs and LA Ɛe by more than 5%, while there was no significant difference in LA Ɛa 16. These findings were like what we observed in the high-dose group, where we found that LA Ɛs was higher in the discontinued treatment group compared to patients currently undergoing treatment. In our study, we observed a significant increase in LA Ɛa in the leukemia group compared to the normal group. Conversely, the high-dose group exhibited a trend towards decreased LA Ɛa relative to the low-dose group, a finding the opposite of LAEF. This trend suggested a potential compensatory increase in LAS in the leukemia patients but this compensatory failed to increase the ejection in reality. As the cumulative dose increases, the LAS subsequently exhibits a decreasing trend. When ventricular diastolic function is compromised, the LA's pumping function may increase to maintain adequate left ventricular filling and cardiac output as a compensatory mechanism (24). However, as the cumulative dose of anthracyclines reaches a certain level, their cardiotoxic effects intensify. Given that atrial myocytes have weaker regenerative capabilities than ventricular myocytes, once damaged, the recovery and repair of atrial myocytes may be more challenging, leading to a decline in the left atrium's ability to sustain compensatory pumping (25, 26). The reasons for the differences in findings compared to those of Fernández-Avilés, Consuelo et al. may be attributed to their selection of participants, who were survivors of childhood ALL completing treatment for over 3 years and being treated higher doses of anthracyclines. On the other hand, LA reservoir strain (Ɛs) measured by CMR feature tracking in our cohort was substantially higher (>70%) than the nominal reference values (∼50%) reported in previous echocardiographic studies (27). We attribute this discrepancy primarily to technical factors: normative CMR-derived atrial strain data for young children (mean age, 7 years in our cohort) remain scarce, and our measurements were obtained using an earlier-generation 1.5 T scanner platform. Therefore, this absolute numerical deviation likely represents a technology-specific offset rather than a pathological finding. Importantly, our dataset provides a valuable supplement to the current body of CMR-derived atrial strain reference values in the pediatric population.

### Changes in left ventricular strain in pediatric leukemia patients with different cumulative dosage groups

In LVS, GLS is a robust and sensitive marker of LV dysfunction in cancer therapy-related cardiac dysfunction (28). Evangelos K. Oikonomou et al. summarized in their meta-analysis that most studies have found that patients receiving higher doses of anthracyclines are more likely to experience a significant reduction in GLS, thereby increasing the risk of Chemotherapy-Induced Cardiotoxicity (CTRCD) (29). In this study, no significant intergroup difference in LVGLS was observed between the overall leukemia cohort and healthy controls. However, subgroup analysis revealed a trend toward reduced LVGLS in the high-dose subgroup compared with controls (*p* = 0.055). Although this did not reach conventional statistical significance, it may warrant attention as a potential signal of dose-related myocardial deformation impairment at a very early time, consistent with previous research.

### Changes in LACI in pediatric leukemia patients with different cumulative dosage groups

LACI (Left atrioventricular coupling index) is a crucial parameter for assessing the physiological connection between the left atrium and left ventricle, reflecting the functional state of the heart at end-diastole, particularly the volume relationship between the atrium and ventricle (30, 31). Studies demonstrated that LACI was independently associated with all-cause mortality and heart failure hospitalization risk in various cardiovascular diseases. Elevated LACI values might indicate a functional imbalance between the left atrium and left ventricle, thereby increasing the risk of cardiac dysfunction (32, 33). This imbalance typically manifested as an increase in left atrial volume and a decrease in left ventricular volume, which aligned with our research findings. We observed higher LACI values in the high-dose group, and as the cumulative dosage of anthracycline drugs increased, so did the LACI, potentially indicating a greater functional imbalance between the left atrium and left ventricle compared to the low-dose group. However, it is noteworthy that the LACI values in both the high-dose and low-dose groups were relatively low in our population, compared to the study by Gizem Kasa et al., which found that patients with LACI values exceeding 30.9% had significantly worse prognosis than others (33). This might be related to the population we selected, or the absolute dosage and type of anthracycline drugs used.

### Discoordination in left atrial and ventricular changes

The left atrium and left ventricle function as an integrated system, and coordination between them is crucial for normal function (34). Our study found that cumulative anthracycline dosage resulted in inconsistent changes in the volumes of the left atrium and left ventricle. Compared to the low-dose group, the diastolic left atrial volume tended to increase, while the left ventricular volume tended to decrease in the high-dose group, like the findings reported by Peter Emerson (35). As a ratio of the left atrium to left ventricle at end-diastole, the LACI value in the high-dose group was greater than that in the low-dose group, indicating a discoordination in the volume relationship between the left atrium and left ventricle at end-diastole in the high-dose group compared to the low-dose group. Additionally, greater cumulative anthracycline dosage led to inconsistent changes in the strains of the left atrium and left ventricle. The research by Malaescu et al. found a strong correlation between LAS and LVGLS (36), which aligned with our observations in normal children. However, in leukemia patients, this correlation was weakened and there were slight variations in the correlation between LAS and LVS among leukemia children receiving different cumulative doses of anthracyclines. We hypothesize that this may be due to the discoordination in left atrial and ventricular strain induced by anthracycline treatment, potentially related to differences in resilience or the timing of damage between the left atrium and left ventricle (25). Apart from the varying correlations between LVS and LAS across different groups, we also observed that in the leukemia group, LAS exhibited an increasing trend compared to the normal group, whereas LVS demonstrated a decreasing trend (although this difference was not statistically significant). However, the inconsistency in volume or functional changes of the left atrium and left ventricle under the influence of anthracycline drugs would require further prospective and larger-scale research for confirmation.

Cardiac dysfunction is a common sequela of anthracycline-induced cardiac toxicity (16). This study targeted a group of children undergoing relatively low toxicity daunorubicin therapy (37) as a cross-sectional observation cohort. Early inconsistencies in left atrial and ventricular dimensions or functions were observed in these children. However, the predictive value of these findings for future cardiac dysfunction or adverse cardiovascular events remains uncertain. Therefore, our research team is continuously conducting rigorous follow-up studies on this cohort of children to further elucidate the relationship between left atrial and ventricular inconsistencies and long-term cardiovascular health.

### Limitations

The cross-sectional design and variable timing of post-treatment CMR (128–177 weeks from initiation) likely explain the non-significant trends in LA and LV strain. Despite a ≤1-year cessation window, this temporal heterogeneity introduces confounding variance. Future studies should standardize CMR timing across chemotherapy phases and incorporate serial assessments during treatment to better capture dose-dependent atrial and ventricular remodeling. This study was a single-center study, future work should expand the population and prolong follow-up to clarify the long-term impact of childhood left ventricular strain changes on survival outcomes. This study did not adjust for inter-group age/BSA differences, reflecting the age/BSA-dependent dosing paradigm in pediatric leukemia where younger children (<10 years) receive lower doses per guidelines (20) (Supplementary Table S5). To address age confounding, we standardized LA and LV volume measurements by BSA, effectively correcting somatic growth variations and improving chemotherapy-related cardiac toxicity assessment accuracy. We acknowledge that inevitable intergroup age differences may confound LA strain measurements. Previous studies (e.g., Boston Children's Hospital; Nijmegen Heart Centre) have demonstrated an age-related decline in strain and EF, paralleling left ventricular function (38, 39). Therefore, the trend of decreased absolute LA and LV strain in the high-dose group (Table 3) might be attributable to age progression rather than solely to increased dosage. To address this, we performed multiple linear regression adjusting for age; results showed that the differences in LA and LV strain between dose groups remained statistically insignificant (Supplementary Table S6). Notably, the unadjusted analysis in Table 3 also showed no statistical significance. Furthermore, our study emphasizes left atrial-ventricular coupling mechanisms rather than dose-strain dependency. In addition, age differences were strictly controlled in the primary comparison between healthy controls and leukemia patients.

## Conclusions

This study, focusing on the population of Chinese children with leukemia, revealed differential effects of varying cumulative doses of anthracyclines on the inconsistent changes in the left atrium and left ventricle in this population. Additionally, it identified an association between left atrial-ventricular synchrony and drug dosage, providing new insights into the search for a compensatory threshold for LACI or left atrial strain.

## Data Availability

The original contributions presented in the study are included in the article/Supplementary Material, further inquiries can be directed to the corresponding author/s.
