# Supplementary material for: Anthracycline-induced left atrioventricular dyssynchrony in children with leukemia: a cardiac magnetic resonance imaging study of functional and strain analysis
Source: Front Cardiovasc Med. 2026 Jul 29;13:1874142. doi: 10.3389/fcvm.2026.1874142 (PMC13461419; doi:10.3389/fcvm.2026.1874142)
Supplement: Supplementary file 1 [file Datasheet1.docx]

# Supplementary Materials

Figure S1 | Flow chart of normal and leukemia children enrolled in this study.

CMR: Cardiac Magnetic Resonance.

**Table S1 | Basic clinical characteristics of the leukemia group and the normal group.**

| **Characteristic** | **group** | | **p-value** |
| --- | --- | --- | --- |
|  | **normal  N = 40^1^** | **leukemia  N = 109^1^** |  |
| **Age at examination** | 9.0 (6.5, 10.0) | 7.3 (5.0, 10.8) | 0.489^2^ |
| **BMI at examination** | 17.0 (15.3, 18.4) | 17.2 (15.7, 19.3) | 0.219^2^ |
| **BSA at examination** | 1.05 ± 0.21 | 0.95 ± 0.30 | 0.020^3^ |
| **Sex** |  |  | 0.403^4^ |
| Male | 24 (60.0%) | 57 (52.3%) |  |
| Female | 16 (40.0%) | 52 (47.7%) |  |
| ^1^Median (Q1, Q3); Mean ± SD; n (%)  ^2^Wilcoxon rank sum test  ^3^Welch Two Sample t-test  ^4^Pearson's Chi-squared test  **BMI:** Body Mass Index, **BSA:** Body Surface Area. | | | |

**Table S2 | Comparison of LAS, LVS and conventional magnetic resonance parameters and strain in the normal, low-dose and high-dose groups.**

| **Characteristic** | **Comparison 1** | | **p-value** | **Comparison 2** | | **p-value** |
| --- | --- | --- | --- | --- | --- | --- |
|  | **low-dose group** | **normal** group |  | **high-dose group** | **normal** group |  |
|  | **N = 83**^1^ | **N = 40**^1^ |  | **N = 26**^1^ | **N = 40**^1^ |  |
| **HR** | 87 ± 15 | 92 ± 15 | 0.079^2^ | 81 ± 19 | 92 ± 15 | **0.017^2^** |
| **LACI (%)** | 8.9 (7.1, 10.9) | 8.4 (6.3, 11.0) | 0.558^3^ | 10.0 (6.4, 12.3) | 8.4 (6.3, 11.0) | 0.502^3^ |
| **LA function and strain parameters** | | | |  | | |
| **LA Ɛs (%)** | 75 (60, 83) | 71 (63, 78) | 0.139^3^ | 67 ± 12 | 69 ± 13 | 0.490^2^ |
| **LA Ɛe* (%)** | 49 (37, 57) | 51 (44, 55) | 0.604^3^ | 44 ± 8 | 49 ± 10 | 0.063^2^ |
| **LA Ɛa*** (%) | 25 (20, 30) | 21 (16, 26) | **0.010^3^** | 23 ± 9 | 21 ± 7 | 0.312^2^ |
| **LAVmax I** | 24 (20, 31) | 29 (25, 32) | **0.049^3^** | 23 ± 9 | 28 ± 7 | **0.033^2^** |
| **LAVmin I** | 6.33 (4.87, 7.78) | 5.59 (4.12, 6.99) | 0.134^3^ | 6.64 (4.50, 9.81) | 5.59 (4.12, 6.99) | 0.311^3^ |
| **LAVpre-A I** | 12.6 (10.1, 15.2) | 10.3 (8.6, 13.4) | **0.047^3^** | 9.8 (7.7, 15.5) | 10.3 (8.6, 13.4) | 0.902^3^ |
| **LA total** (%) | 73 ± 8 | 78 ± 7 | **<0.001**^2^ | 71 ± 9 | 78 ± 7 | **<0.001**^2^ |
| **LA passive** (%) | 49 (40, 56) | 59 (53, 65) | **<0.001**^3^ | 52 (39, 61) | 59 (53, 65) | **0.015^3^** |
| **LA active** (%) | 49 (39, 57) | 50 (40, 58) | 0.985^3^ | 39 ± 16 | 48 ± 13 | **0.020^2^** |
| **LV** function and strain parameters | | | |  | | |
| **LVEDVI** | 72 (60, 79) | 67 (59, 76) | 0.118^3^ | 68 ± 11 | 66 ± 12 | 0.430^2^ |
| **LVESVI** | 25.1 (20.5, 29.3) | 24.6 (20.8, 28.6) | 0.703^3^ | 25.3 ± 5.2 | 24.7 ± 5.4 | 0.648^2^ |
| **LVSVI** | 46 (40, 51) | 42 (35, 48) | **0.025^3^** | 43 ± 8 | 41 ± 9 | 0.449^2^ |
| **LVCOI** | 3.81 (3.37, 4.35) | 3.76 (3.24, 4.27) | 0.501^3^ | 3.43 ± 0.79 | 3.75 ± 0.87 | 0.131^2^ |
| **LVEF(%)** | 64.7 ± 5.1 | 62.4 ± 5.7 | **0.032^2^** | 63.7 ± 6.5 | 62.4 ± 5.7 | 0.397^2^ |
| **LVGLS* (%)** | 19.55 (18.07, 21.08) | 20.50 (18.06, 21.12) | 0.495^3^ | 17.94 (15.94, 21.14) | 20.50 (18.06, 21.12) | 0.055^4^ |
| **LVGRS (%)** | 37 (34, 41) | 38 (30, 41) | 0.707^3^ | 34 ± 8 | 37 ± 7 | 0.276^2^ |
| **LVGCS* (%)** | 19.91 ± 1.82 | 20.08 ± 1.57 | 0.607^2^ | 19.85 ± 2.02 | 20.08 ± 1.57 | 0.629^2^ |
| ^1^Median (Q1, Q3); Mean ± SD | | | |  | | |
| ^2^Wilcoxon rank sum test | | | |  | | |
| ^3^Welch Two Sample t-test | | | |  | | |
| ^*^ For the values of LA Ɛe, LA Ɛa, LVGLS and LVGCS, take their absolute values. | | | |  | | |
| Abbreviations as in Table 2. | | | |  | | |

| **Characteristic** | **Univariable** | | | | **Multivariable** | | | |
| --- | --- | --- | --- | --- | --- | --- | --- | --- |
|  | **N** | **Beta** | **95% CI** | **p-value** | **N** | **Beta** | **95% CI** | **p-value** |
| **group** |  |  |  |  |  |  |  |  |
| **high-dose group** | 26 | — | — |  | 26 | — | — |  |
| **low-dose group** | 83 | 8.57 | 2.20, 14.94 | 0.010 | 83 | 7.10 | -0.35, 14.56 | 0.065 |
| **log (Age at examination)** | 109 | -5.78 | -11.51, -0.05 | 0.051 | 109 | -2.52 | -9.14, 4.09 | 0.456 |
| Abbreviation: CI = Confidence Interval | | | | | | | | |
|  | | | | | | | | |

**Table S3 | Univariable and multivariate linear regression analysis of log (Age at examination) and dose groups for the LA active (%) in leukemia patients.**

Table S4 | Comparison of LAS, LVS and conventional magnetic resonance parameters in different phase of treatment with different dosage groups in leukemia patients.

| **Characteristic** | **low-dose group, N = 83** | | | **high-dose group, N = 26** | | |
| --- | --- | --- | --- | --- | --- | --- |
|  | **Under medication,**  **N = 63^1^** | **Drug discontinuance,**  **N = 20^1^** | **p-value^2^** | **Under medication,**  **N = 19^1^** | **Drug discontinuance,**  **N = 7^1^** | **p-value^3^** |
| **Ɛs (%)** | 75 (65, 82) | 75 (58, 83) | 0.941 | 59 (57, 71) | 78 (69, 82) | **0.024** |
| **Ɛe* (%)** | 49 (37, 57) | 48 (38, 56) | 0.840 | 42 (38, 48) | 46 (45, 52) | 0.118 |
| **Ɛa* (%)** | 25 (20, 30) | 23 (15, 29) | 0.391 | 21 (15, 26) | 23 (21, 34) | 0.169 |
| **max I** | 24 (20, 30) | 24 (21, 31) | 0.737 | 23 (19, 30) | 17 (17, 19) | 0.107 |
| **min I** | 5.99 (4.80, 7.46) | 6.81 (5.54, 7.85) | 0.346 | 6.96 (5.31, 10.05) | 4.13 (2.72, 5.91) | **0.018** |
| **pre-A I** | 12.4 (10.2, 15.9) | 12.8 (10.7, 14.4) | 0.945 | 13.4 (9.5, 15.7) | 7.7 (5.6, 9.6) | 0.063 |
| **LA total** | 74 (70, 77) | 74 (69, 77) | 0.802 | 71 (63, 73) | 75 (73, 82) | **0.030** |
| **LA passive** | 49 (38, 55) | 51 (43, 57) | 0.330 | 50 (40, 56) | 54 (43, 67) | 0.364 |
| **LA active** | 49 ± 13 | 43 ± 16 | 0.110 | 37 ± 13 | 45 ± 22 | 0.449 |
| **LVEDVI** | 71 (60, 76) | 75 (64, 84) | 0.154 | 69 (62, 77) | 68 (66, 69) | 0.707 |
| **LVESVI** | 24.7 (20.4, 29.0) | 27.4 (23.8, 30.3) | 0.115 | 25.9 (23.0, 29.0) | 22.7 (21.9, 24.1) | 0.157 |
| **LVSVI** | 45 (39, 51) | 48 (42, 52) | 0.431 | 42 (37, 50) | 45 (41, 46) | 0.707 |
| **LVCO** | 3.23 (2.56, 3.91) | 3.27 (2.38, 4.07) | 0.979 | 4.14 (3.59, 5.07) | 3.99 (3.72, 4.51) | 0.583 |
| **LVEF** | 65.3 ± 5.3 | 63.0 ± 4.1 | 0.058 | 63 ± 6 | 67 ± 8 | 0.240 |
| **LVGLS* (%)** | 19.61 (18.90, 21.11) | 19.46 (17.96, 20.22) | 0.636 | 18.09 (15.81, 21.09) | 17.79 (16.97, 20.74) | 0.583 |
| **LVGRS (%)** | 37 (34, 41) | 37 (34, 41) | 0.970 | 34 (26, 42) | 34 (30, 40) | 0.840 |
| **LVGCS* (%)** | 20.03 ± 1.85 | 19.55 ± 1.69 | 0.291 | 19.69 ± 1.95 | 20.28 ± 2.31 | 0.560 |
| **LACI (%)** | 8.7 (7.1, 11.0) | 9.3 (7.7, 10.5) | 0.628 | 10.7 (8.1, 14.2) | 6.1 (4.0, 9.3) | **0.013** |
| ^1^Median (IQR); Mean ± SD | | | | | | |
| ^2^Wilcoxon rank sum test; Welch Two Sample t-test | | | | | | |
| ^3^Wilcoxon rank sum exact test; Wilcoxon rank sum test; Welch Two Sample t-test  ^*^ For the values of LA Ɛe, LA Ɛa, LVGLS and LVGCS, take their absolute values.  Abbreviations as in Table 2. | | | | | | |

Figure S2 | Spearman correlation of LAS and LVS in high-dose group with different treatment phase.

(A) Spearman correlation of LAS and LVS in children under medication. **(B)** Spearman correlation of LAS and LVS in children under drug discontinuance.

*p < 0.05, **p < 0.01, ***p < 0.001.

Table S4 | Intra- and Inter-Observer Variability of LAS and LVS.

|  | **Intraclass Correlation Coefficient (95% CI)** | |
| --- | --- | --- |
|  | **Intra-Observer** | **Inter-Observer** |
| **LAS** |  | |
| εs (%) | 0.905 (0.811-0.954) | 0.932 (0.843-0.962) |
| εe (%) | 0.850 (0.707-0.926) | 0.873 (0.746-0.938) |
| εa (%) | 0.970 (0.939-0.986) | 0.980 (0.959-0.990) |
| **LVS** |  | |
| GLS* (%) | 0.757 (0.548-0.877) | 0.952 (0.902-0.977) |
| GRS (%) | 0.933 (0.865-0.968) | 0.757 (0.605-0.859) |
| GCS* (%) | 0.887 (0.777-0.945) | 0.761 (0.659-0.878) |

^*^ For the values of LVGLS and LVGCS, take their absolute values.

Abbreviations as in Table 4.

Figure S3 | Bland-Altman plots for LAS parameters.

Figure S3 | Bland-Altman plots for LVS parameters.

Table S5 | Univariate linear regression analysis of clinical characteristics for the **cumulative dose of anthracyclines** in leukemia patients.

| **Characteristic** | **N** | **Beta** | **95% CI** | **p-value** |
| --- | --- | --- | --- | --- |
| **Age at examination** | 109 | 0.18 | 0.16, 0.19 | **<0.001** |
| **BMI at examination** | 109 | -0.02 | -0.04, 0.00 | 0.107 |
| **BSA at examination** | 109 | 2.32 | 2.20, 2.44 | **<0.001** |
| CI = Confidence Interval | | | | |
| **BMI:** Body Mass Index, **BSA**: Body Surface Area.  Dependent variable was subjected to logarithmic transformation. | | | | |

**Table S6 | Univariable and multivariate linear regression analysis of log (Age at examination) and dose groups for the LAS and LVS in leukemia patients.**

| **Characteristic** | | **Univariable** | | | | **Multivariable** | | | |
| --- | --- | --- | --- | --- | --- | --- | --- | --- | --- |
|  |  | **N** | **Beta** | **95% CI** | **p-value** | **N** | **Beta** | **95% CI** | **p-value** |
| **LA Ɛs (%)** | **log (Age at examination)** | 109 | -2.72 | -7.86, 2.42 | 0.302 | 109 | -0.39 | -6.36, 5.57 | 0.897 |
|  | **group** |  |  |  |  |  |  |  |  |
|  | high-dose group | 26 | — | — |  | 26 | — | — |  |
|  | low-dose group | 83 | 5.30 | -0.43, 11.03 | 0.073 | 83 | 5.07 | -1.66, 11.79 | 0.143 |
| **LA Ɛe* (%)** | **log (Age at examination)** | 109 | 2.22 | -2.14, 6.57 | 0.320 | 109 | 4.73 | -0.29, 9.76 | 0.068 |
|  | **group** |  |  |  |  |  |  |  |  |
|  | high-dose group | 26 | — | — |  | 26 | — | — |  |
|  | low-dose group | 83 | 2.73 | -2.17, 7.63 | 0.278 | 83 | 5.48 | -0.18, 11.15 | 0.060 |
| **LA Ɛa* (%)** | **log (Age at examination)** | 109 | -4.94 | -8.36, -1.51 | 0.006 | 109 | -5.13 | -9.15, -1.11 | 0.014 |
|  | **group** |  |  |  |  |  |  |  |  |
|  | high-dose group | 26 | — | — |  | 26 | — | — |  |
|  | low-dose group | 83 | 2.57 | -1.40, 6.54 | 0.208 | 83 | -0.42 | -4.95, 4.11 | 0.857 |
| **LVGLS* (%)** | **log (Age at examination)** | 109 | -0.75 | -1.79, 0.30 | 0.165 | 109 | -0.25 | -1.47, 0.96 | 0.684 |
|  | **group** |  |  |  |  |  |  |  |  |
|  | high-dose group | 26 | — | — |  | 26 | — | — |  |
|  | low-dose group | 83 | 1.22 | 0.06, 2.39 | 0.042 | 83 | 1.08 | -0.29, 2.45 | 0.126 |
| **LVGRS (%)** | **log (Age at examination)** | 109 | -1.31 | -4.77, 2.15 | 0.460 | 109 | 0.36 | -3.66, 4.37 | 0.862 |
|  | **group** |  |  |  |  |  |  |  |  |
|  | high-dose group | 26 | — | — |  | 26 | — | — |  |
|  | low-dose group | 83 | 3.43 | -0.43, 7.28 | 0.084 | 83 | 3.63 | -0.89, 8.15 | 0.118 |
| **LVGCS* (%)** | **log (Age at examination)** | 109 | -0.09 | -0.82, 0.64 | 0.816 | 109 | -0.08 | -0.94, 0.78 | 0.857 |
|  | **group** |  |  |  |  |  |  |  |  |
|  | high-dose group | 26 | — | — |  | 26 | — | — |  |
|  | low-dose group | 83 | 0.06 | -0.76, 0.88 | 0.881 | 83 | 0.02 | -0.95, 0.98 | 0.973 |
| CI: Confidence Interval, **LA Ɛs** : reservoir strain. **LA Ɛe** : conduit strain. **LA Ɛa** : contraction strain.**LVGLS** : global longitudinal strain. **LVGRS**: global radial strain. **LVGCS**: global circumferential strain.  *For the values of LA Ɛe, LA Ɛa, LVGLS and LVGCS, take their absolute values. | | | | | | | | | |
